# Some Considerations in Roofing

**Published:** 1919-10-25

**Authors:** 


### HOSPITAL CONSTRUCTION: THE NEW ERA.

### XII. Some Considerations in Roofing.

A recent article in this series has brought us some correspondence. A writer interested in concrete construction has written in defence of the material, and points out that our objection to ferro-concrete as not lending itself readily to alterations after completion can be met by the use of concrete blocks or concrete slabs—in other words, by the abandonment of the reinforcement system.

The writer of the letter frankly admits that concrete cannot "have it both ways." If we accept the almost miraculous benefit of reinforcement we must give up the convenience of easy alteration; if, on the other hand, we choose to use concrete in the block form—either as artificial stone or as partition slabs—we lose the strength and the homogeneity which characterise ferro-concrete. It is further pointed out that the ease with which rounded angles can be formed in concrete construction gives it a further claim on our consideration as a hospital material.

On the subject of condensation this advocate of concrete has some recommendations to offer. He suggests the use of the expedient to which concrete builders have already had recourse. This consists of a double device; a cavity and a porous inner wall. We have every reason to suppose that this remedy has proved more or less successful in checking the display of fluid on the inner surface of the wall. In other words, it substitutes saturation for surface liquification. Whether this is an advantage, except from the point of view of appearances, is still open to doubt. In any case it is plain that concrete and ferro-concrete have to be taken with an antidote. We have in using them to purchase two forces which cancel one another, and unless concrete can show a bill of costs so vastly below the price of materials that need no such antidotes as to win outright in spite of the handicap, it will necessarily go hard with concrete in the race for economy.

Our discussion of walls leads very naturally to some consideration of roofs. Here again the battle of prices rages very fiercely. Among the permanent materials a really ugly slate is an easy winner. It costs as a rule less than tiles and it can be laid at a much lower pitch. This latter quality is of great economic value. The lower the pitch, the smaller the area of the roofing itself, so there to begin with is a direct saving in the superficies of material required, and this applies of course not merely to the roof covering itself but also to the supporting timbers, rafters and battens, and to the boarding, if employed—and this is not all. The flatter the pitch employed, the less waste of space will there be in the roof apex above the level of the topmost ceiling.

Of lead and copper there is no need to speak in the same breath as the word "economy." They may in these unhappy days be written off as luxuries. But we have to consider before talking of the "temporary" materials the claims of asphalte and other bituminous coverings. There is no reason in the nature of things why asphalte should not be the perfect roof. We have in asphalte a material of unquestionable waterproofness, capable of being laid in very thin layers and able to throw off water at a very slight gradient. A flat roof is necessarily or frequently surrounded by parapets, and these parapets have to be coated to a certain height with waterproof aprons or flashings to prevent the percolation of the surface water down the walls. Here again asphalte is naturally fitted to meet the case, for its ductile surface can be turned up against the walls in a sheet homogeneous with the flat itself. In point of fact acres of roof have been made in this way and have stood the test of time and weather.

But there is nothing to be gained by denying the fact that asphalte is desperately treacherous. A certain London architect is ready to assert after thirty years of general practice that he will never willingly submit himself again to the anxieties which attend the erection of an asphalte roof-covering. He can point, he says, to roof after roof which has "let him down."

Sometimes the asphalte layers when appealed to after a failure attribute the leakages to settlements in the building. But this is no good excuse, for asphalte should of its very nature have sufficient elasticity to bend rather than break when any disturbance of its bed takes place. The fact is that for some reason or other asphalte has a trick of being either softer or harder than it ought to be. Asphalte used in a damp-proof course has been known to ooze from its bed so freely as to displace the wall above it, and again in roofs it will sometimes be so hard as to crack like glass. It is this horrible uncertainty that makes the use of asphalte a gamble. Far better than asphalte is the material "Vulcanite"—a bituminous substance laid in layers with felt. It needs to be protected from the action of the sun and the makers recommend a coating of loose gravel, but in practice it is found preferable to cover it with a coating of thin breeze concrete, just thick enough and just strong enough to hold together. For buildings of a definitely temporary character there are various types of felt bituminised in different degrees, most of which are better laid on a slight slope than on a flat surface.

It remains to say something of asbestos slates. There are various forms of these on the market. Some are tinted red to resemble ordinary pottery tiles, but the resemblance never amounts to successful deception. Others are left in the grey tone, which is the natural colour of the material. They are all undeniably ugly, and the prevalent method of hanging them—*viz.*, diagonally with a point at top and bottom—increases their unorthodox appear-

**Previous articles appeared on Jan. 25, Feb. 8, March 1. 15, 29, April 19, May 17, June 14, July 19, Aug. 23, and Sept. 20, p. 613**

**Hospital Construction: The New Era—(*continued*).**

ance. Unhappily for those who like good looks in a building, there is a great deal to be said for this material, whether it be called asbestos, uralite, or eternit. It is definitely wet-resisting, it can be laid with the minimum of lap or cover, and it is so surprisingly light as to call for much less strength in the roof timbers than other materials require.

It cannot be laid flat of course; but it can be laid somewhat flatter than ordinary tiles. Probably the best way of employing this covering where appearance is of importance would be to confine its use to low-pitched roofs concealed behind parapets. Perhaps its length of life has not yet been fully tested, but the present writer has reason to believe from a personal experiment that its duration is very great. The material, by the way, if used in the form of slabs, not as tiles, is a very good device for the inner protection of damp walls. An ancient farm house of the pre-damp-course period had its old-fashioned hall panelled eight or ten years ago with eternit set in styles and rails of woodwork. The expedient proved quite sufficient to keep all visible traces of damp in the background. It may be argued that this was only an *apparent* cure of the mischief, but after all every brick wall is wet on the outside and generally wet for some distance inwards.

